# Guide Extension-Facilitated Ostial Stenting (GEST) During Primary Coronary Angioplasty in a Patient With ST-Segment Elevation Myocardial Infarction: A Case Report

**DOI:** 10.7759/cureus.75148

**Published:** 2024-12-05

**Authors:** George Besis, Zahid Khan, Nay Thu Win, Muscab Soyan, Luciano Candilio

**Affiliations:** 1 Cardiology, Royal Free Hospital, London, GBR; 2 Acute Medicine, Mid and South Essex NHS Foundation Trust, Southend-on-Sea, GBR; 3 Cardiology, Bart’s Heart Centre, London, GBR; 4 Cardiology and General Medicine, Barking, Havering and Redbridge University Hospitals NHS Trust, London, GBR

**Keywords:** acute st-elevation myocardial infarction, coronary angioplasty with stenting, deep coronary intubation, guide extension catheter, guide extension-facilitated ostial stenting (gest), primary pci, primary percutaneous coronary intervention (pci), right coronary artery (rca), severe coronary artery disease, tortuous coronary anatomy

## Abstract

The guide extension-facilitated ostial stenting (GEST) technique uses a guide extension catheter (GEC) to improve stent delivery during primary coronary angioplasty (PCI). GECs are used for stent delivery into the coronary arteries of patients with difficult anatomy due to tortuosity, calcification, or chronic total occlusion (CTO) vessels. Stent and balloon placement has become challenging in patients with increasing lesion complexity due to tortuosity, vessel morphology, length of the lesion, and respiratory movements. To overcome this challenge, a guide catheter can be useful support enabling the deployment of stents in such challenging cases. We present the case of a 56-year-old male patient who was brought in by an ambulance with out-of-hospital ventricular fibrillation arrest (OOHVFA) and return of spontaneous circulation (ROSC) following successful resuscitation. The patient underwent emergency coronary angioplasty, which revealed extensive disease in the left coronary arteries and severe disease in the right coronary artery (RCA). Coronary angioplasty proved challenging secondary to vessel tortuosity, and a GEC was used for stent placement. Two stents were placed into the RCA in an overlapping fashion with the help of GEC to achieve thrombolysis in myocardial infarction (TIMI-3) flow. The patient was admitted to the intensive care unit (ICU), and echocardiography revealed mild-to-moderate left ventricular systolic dysfunction. The patient required significant inotropic support, and a computerised tomography (CT) scan of the brain showed severe hypoxic encephalopathy. The patient did not show any improvement and remained under intubation. The patient was extubated following a discussion with his family and palliative care team and passed away peacefully.

## Introduction

Primary coronary angioplasty (PCI) can be challenging in patients with calcific and tortuous vessels because of poor lesion compliance and difficulty in stent delivery [1]. Optimal stent delivery remains a significant challenge for patients undergoing coronary angioplasty [1]. Various techniques have been used to optimise stent delivery and implantation, such as vessel straightening using buddy and support wires, high-pressure dilatation to prepare vessels and increase catheter support during the procedure through deep intubation, use of guide extension catheters (GECs), and anchor balloon techniques [1]. de Man et al. reported a single-centre study based on a large cohort of patients who underwent PCI performed by senior operators with the aid of a 5 Fr compatible GuideLiner catheter (Vascular Solutions Inc., Minneapolis, MN, US) in a 6 Fr catheter system. The indications for using GuideLiner support during angioplasty in these patients included backup support during stent delivery in 41 cases, selective contrast injection in 20 cases, and improved catheter alignment in 20 cases [2]. The device success rate was 93% in this study, and no major complications were reported.

A major advantage of GEC is that it can be used to advance across the points of resistance or tortuosity in the proximal vessel, allowing delivery of a stent within the catheter directly to the mid or even distal vessel with little or no resistance [3,4]. Another key advantage of GECs is the selective contrast injection, as reported by de Man et al., that deep intubation achieved with GECs allows direct contrast delivery to the target lesion requiring a smaller amount of contrast, and greater opacification can be achieved. This is particularly important in patients with renal failure and opacification of the dual native and graft vessels [1,2]. Liao et al. conducted a single-centre study in patients undergoing complex coronary angioplasty and reported that 5-4 Fr Expressman GECs (APT, China) were superior to 5 Fr GECs [5]. Despite their usefulness, these catheters also have some limitations, including difficulty in advancing them and the potential risk of vessel injury associated with them [6,7]. It is worth mentioning that the shape and size of the GEC have an impact on the passive and active backup support function provided by it. The former is dependent on the physical characteristics of the GEC wall such as stiffness of the GEC and distal preformed shape, which allows the ability of the GEC to achieve coaxial alignment within the coronary ostium allowing it to rest for support on the opposite of the aorta or the aortic valve. The active support is achieved by operator-dependent techniques such as GEC manipulation for deep seating engagement or coaxial alignment, and the smaller catheters such as 5 and 6 Fr offer the greater advantage of allowing deeper intubation compared to larger catheters [8]. GEC allows operators greater capability to deliver stents and balloons by providing 5-15 cm extra depth beyond that of a traditional guide catheter. Herein, we present a case of complex PCI requiring the use of GECs for stent delivery to achieve optimal angiographic results.

## Case presentation

A 56-year-old man with no medical history experienced a sudden collapse at home. Immediate bystander cardiopulmonary resuscitation (CPR) was commenced by his wife. The ambulance crew that attended the scene found him in ventricular fibrillation, and a total of 11 shocks were administered to achieve a sustained return of spontaneous circulation (ROSC). He received two doses of amiodarone and five doses of adrenaline according to the guidelines of the United Kingdom Resus Council. Immediate post-ROSC electrocardiogram (ECG) showed inferior ST-segment elevation, and the patient was transferred to our centre for primary percutaneous coronary intervention.

On arrival, the patient was intubated and had inferior ST-segment elevation. The patient was immediately transferred to the catheterisation laboratory for an emergency coronary angioplasty. The procedure was performed using a right radial approach. The left system had extensive disease of the left anterior descending and circumflex arteries. The right coronary artery (RCA) had severe disease, extending from the proximal to the middle part of the vessel. Owing to vessel tortuosity, guide extension was used to deliver the first stent in the middle part of the RCA. After stent optimisation, we proceeded with the placement of a second stent in an overlapping fashion with the first stent, covering the RCA ostium. To reduce the parallax to the degree possible, we used a modified spider view. Initial attempts to accurately position the stent in the RCA ostium were hampered by the excessive movement of the stent with respiration and cardiac motion. To reduce the effect of these two factors, we first used a guide extension to confirm the ostial position of the stent and subsequently deployed the stent in the optimal position. Intravascular imaging after stent deployment confirmed an accurate stent placement covering the ostium of the RCA (Videos 1-3).

**Video 1:** The guide extension-facilitated ostial stenting (GEST) technique can be equally useful when smaller stents are deployed in the RCA ostium. Note the excessive movement at the level of the crux and contrast that with the position of the stent in the ostium of the vessel. The stent remains in the same position at the RCA ostium during all the phases of the cardiac cycle allowing for an accurate deployment. RCA: right coronary artery

**Video 2:** Primary coronary angioplasty to the right coronary artery using guide extension for stent deployment to the ostial lesion.

**Video 3:** Intravascular ultrasound imaging during coronary angioplasty showing optimal stent apposition and expansion.

**Figure 1:**
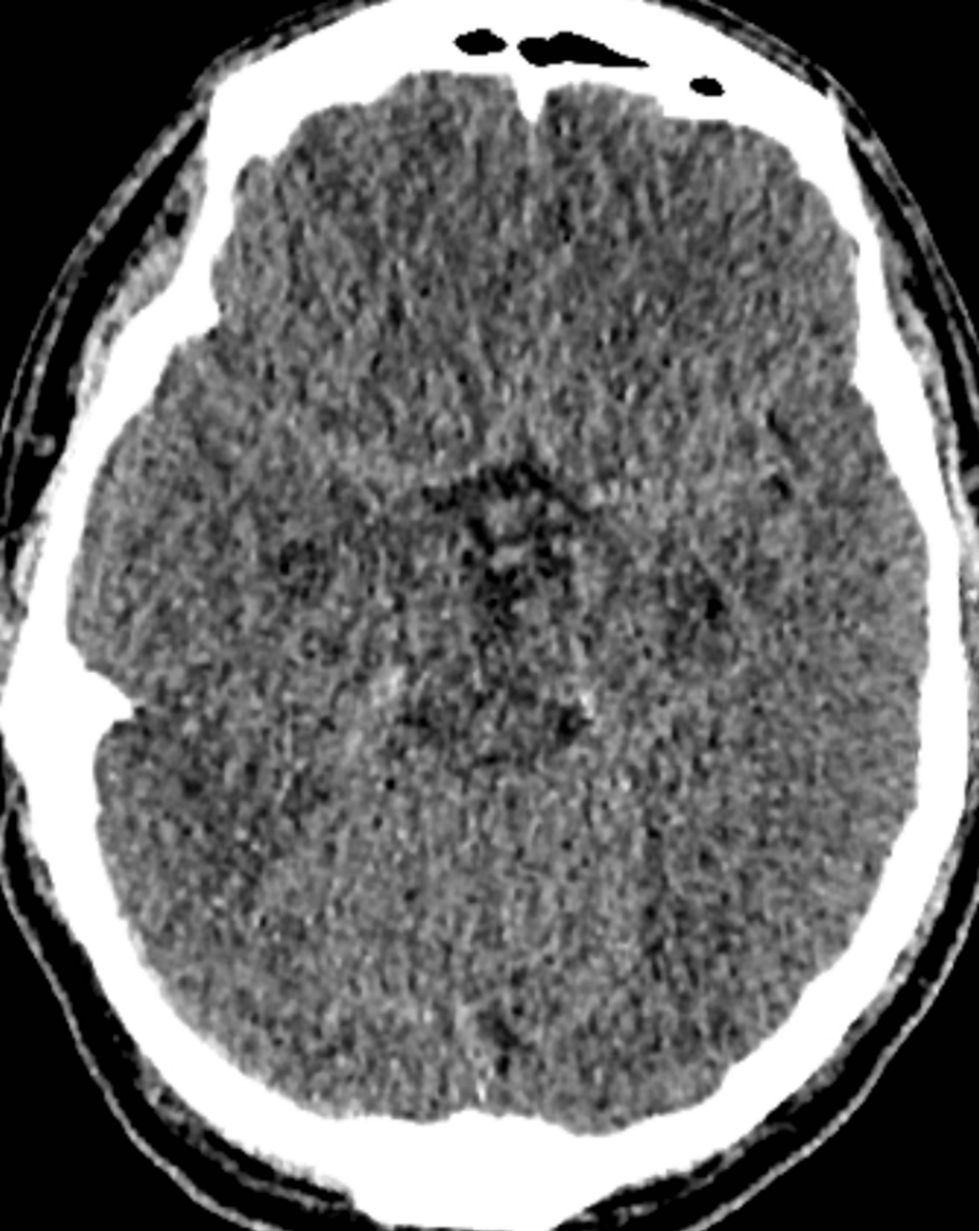
Computerised tomography scan of the brain showing hypoxic ischaemic brain encephalopathy features of decreased cortical grey matter attenuation with a loss of normal grey-white differentiation and decreased bilateral basal ganglia attenuation.

The patient was intubated and admitted to the intensive care unit (ICU), requiring inotropic support. A computerised tomography (CT) scan of the brain 48 hours post-admission showed severe hypoxic brain encephalopathy (Figure 1). Echocardiography in the ICU showed mild left ventricular systolic dysfunction, with a left ventricular ejection fraction of 45%-50% and inferior wall hypokinesia (Videos 4, 5). The patient remained intubated and showed no significant improvement. The prognosis was deemed poor, and consultations were held with the family and palliative care teams. Unfortunately, the patient succumbed to this illness a few days after hospital admission.

**Video 4:** Parasternal long-axis view echocardiography showing mild left ventricular systolic dysfunction.

**Video 5:** Apical four-chamber view echocardiography showing mild left ventricular systolic dysfunction.

## Discussion

Optimal visualisation of the coronary ostium requires projections in which the ostium is depicted in an orthogonal view. In determining optimal projections, useful lessons can be drawn from the structural field. Accurate placement of a transcatheter aortic valve is aided by pre-procedurally determined optimal C-arm projections derived from a dedicated CT scan. These views were selected to avoid parallax when visualising the aortic valve orifice. In this process, the S-curve plays a central role. Introduced by Piazza et al., the S-curve was derived by applying mathematical formulas in an ECG-gated CT scan [8]. Every point on the S-curve corresponds to the angulations that produce an orthogonal view of the aortic valve. When the same principle is applied with a focus not on the aortic valve but on the coronary ostia, the respective S-curves can be calculated. The issue that arises is that the angulations derived by this method are, in a significant proportion of cases, impractical to achieve in the Cath lab. This is particularly true for the RCA, where the optimal angulations are in the range of LAO 64 and CAUD 51 [8,9].

Targoński et al.'s findings were similar to the optimal angulations for the right coronary ostium between LAO 60 and LAO 80 with slight cranial angulation [10]. The most practical solution to optimally visualise a coronary ostium without parallax is to align the catheter with the ostium and select a view that depicts the tip of the catheter as circular and the contour of its proximal part as a set of straight lines. Several techniques have been developed to determine the exact location of the coronary ostium. They can be divided into techniques that provide direct visualisation of the ostium and methods that indirectly determine the exact location of the coronary ostium [11,12]. Intravascular ultrasound (IVUS) has been used for the real-time imaging of the ostium.

Indirect methods to determine the location of the coronary ostium include the floating wire and floating snare techniques. A hybrid approach was also described using a floating wire technique, in which an IVUS probe was mounted [13]. On the other hand, the Szabo technique should be considered a separate approach to the ostial stenting challenge, as the stent is manipulated before entering the guide catheter so that the proximal stent edge can play the role of a proximal marker for the ostium location by placing a coronary wire through the proximal stent strut [14]. Although all of these techniques provide a way of determining the location of the coronary ostium, optimal stent placement also requires mitigation of the excessive movement of the equipment in the aorta-ostial area during the final stage of stent deployment. To address this, techniques with rapid pacing during stent deployment have been described. Therefore, it is beneficial to develop an approach by which both accurate identification of the coronary ostium and precise stent placement can be achieved. This paper describes a simple approach. The guide extension helps not only mitigate excessive movement but also via contrast injection to identify the location of the ostium. If the anatomy allows, the ostium can be identified with minimal or no parallax by ensuring the coaxial engagement of the guide extension with the circular appearance of its tip, as previously described. If the angulations required for optimal visualisation are impractical to achieve with C-arm rotation, the introduction of a floating wire through the guide extension can facilitate the location of the coronary ostium.

GECs have become the mainstay of coronary intervention by facilitating device delivery and are used in almost 18% of PCI cases [3,9]. GEC enables smooth equipment stents and balloon delivery in complex and tortuous lesions and improves the coaxial alignment and backup support of the guide catheter. It also allows selective contrast injections and can help to improve the optical coherence tomography imaging quality by ensuring adequate blood clearance [8]. GECs are not without risks, which include coronary dissection, air embolism, wire and pushrod wrapping, and ischaemia [9,15-17].

## Conclusions

Stent positioning in the ostium of a coronary artery poses a challenge, as its accurate placement depends not only on optimal visualisation of the origin of the coronary artery but also on avoiding excessive movement during the final stage of deployment. Guide extension can be an option that addresses both these considerations to achieve accurate stent placement.
